# Investigating Students' Metacognitive Experiences: Insights From the English as a Foreign Language Learners' Writing Metacognitive Experiences Questionnaire (EFLLWMEQ)

**DOI:** 10.3389/fpsyg.2021.744842

**Published:** 2021-08-30

**Authors:** Qiyu Sun, Lawrence Jun Zhang, Susan Carter

**Affiliations:** Faculty of Education and Social Work, The University of Auckland, Auckland, New Zealand

**Keywords:** metacognitive experiences, metacognition, EFL writing, EFL learners, development and validation of questionnaire

## Abstract

While research on metacognitive knowledge and metacognitive strategies in second language (L2) writing has proliferated, little attention has been paid to metacognitive experiences in learning to write. This study contributes a novel 6-point Likert scale questionnaire, *EFL Learners' Writing Metacognitive Experiences Questionnaire (EFLLWMEQ)*, and reports insights into learners' metacognitive experiences gathered from its use. The questionnaire was designed to investigate, first, the nature of students' metacognitive experiences when they learn to write in English as a foreign language (EFL) and, secondly, the relationship between students' metacognitive experiences and their writing performance. To this end, the questionnaire was developed and validated with two independent samples of 340 and 540 Chinese undergraduates whose metacognitive experiences were measured as they learned to write in EFL. Data were subjected to exploratory factor analysis (EFA) and confirmatory factor analysis (CFA), respectively. Findings of EFA and CFA revealed a four-factor structure of students' metacognitive experiences of EFL writing: Metacognitive estimates, metacognitive feelings, online task-specific metacognitive knowledge, and online task-specific metacognitive strategies. Results showed that students' metacognitive experiences had positive correlations with their EFL writing test scores. Importantly, the CFA results from the sample of 540 students supported the four-factor correlated model with the best model fit, confirming the validity and reliability of the *EFLLWMEQ*. This study has theoretical and pedagogical implications for how learners' metacognitive experiences can be managed in learning to write, particularly in EFL classrooms.

## Introduction

Writing is generally regarded as a problem-solving process that requires metacognitive control of text generation and recursive revision, not only in first language (L1) contexts (Hayes, 2012), but also in second language (L2) settings (Hyland and Hyland, 2019). Given that L2 writing entails complex and recursive interaction of cognitive, metacognitive, and affective processes, learners improve their writing performance by developing metacognitive competencies. Researchers have endeavored to improve learners' L2 writing performance by identifying common learner characteristics and innovative pedagogical approaches (e.g., Teng and Zhang, 2016, 2020; Lee and Mak, 2018; Wei et al., 2020; Yu, 2020). Nevertheless, L2 writing remains a daunting task for language learners who struggle to gain metacognitive control.

Metacognition is deemed a crucial determinant for language learning success (Wenden, 1998; Zhang, 2001, 2010; Zhang and Zhang, 2019; Zhang et al., 2019, 2021a). “Metacognition,” initially coined by Flavell (1976), refers to “one's knowledge concerning one's own cognitive processes and products or anything related to them” (p. 232), knowledge that can be used by learners to monitor, regulate and develop their cognitive processes. Metacognition plays a pivotal role in learning to write: “metacognitive variables play an even more important role than linguistic competence in successful L2 writing” (Devine, 1993, p. 116). Therefore, we propose that understanding the relationship between metacognition and writing performance will offer new insights into L2 writing instruction.

Metacognition can be categorized into three subcategories, namely, metacognitive knowledge, metacognitive experiences, and metacognitive strategies (Flavell, 1979; Papaleontious-Louca, 2008; Efklides, 2009). Given that writing is a cognitively demanding process, learners' orchestration of metacognitive knowledge, metacognitive experiences, and metacognitive strategies could help them to monitor and regulate their writing process (Hacker et al., 2009; Wu, 2021). Of the three subcategories, metacognitive knowledge is the foundation that promotes the use of cognitive and metacognitive strategies in the learning process; metacognitive experiences, in turn, instigate the revision of metacognitive knowledge (Garner, 1987; Papaleontious-Louca, 2008). Metacognitive strategies involving planning, monitoring, and evaluating their writing enable learners to achieve their learning goals. A number of studies have investigated the impact of metacognitive knowledge and metacognitive strategies on L2 learners' writing performance (e.g., Zhang, 2008; Ruan, 2014; Zhang and Qin, 2018; Teng and Zhang, 2021; Zhang et al., 2021b. However, research on metacognitive experiences in L2 writing has not gained much attention, despite L2 writing being a common experience for millions of learners. For Chinese EFL learners, EFL writing instruction focuses on passing exams at the expense of active learning (Yang and Gao, 2013, Yu, 2020; Chen et al., 2021), which might impact their metacognitive experiences in learning to writing. This study focuses on the often-overlooked dimension of metacognitive experiences.

Metacognitive experiences, the focus of investigation in this article, include cognitive experiences and affective experiences in the cognitive process (Flavell, 1979). What distinguishes metacognitive experiences from other kinds of experiences is that they involve current and ongoing cognition and emotions, i.e., the affective feelings, involved during the cognitive process (Papaleontious-Louca, 2008). The affective perspective has been increasingly recognized as influential in metacognition and learning (Fisher, 2018). As Prior (2019) states, it seems that, following on from the proverb about blind men experiencing an elephant differently, *elephants* are a popular metaphor in discussions of the emotion about L2 learning. Perhaps this powerful metaphor is employed because emotions vary as much as an elephant's tail differs from its ear, and there is a lack of L2 writing research that makes comprehensive sense of L2 learning emotions. While L2 writing researchers have exclusively investigated either metacognition or emotion (e.g., Ruan, 2014; Jin and Zhang, 2021), little attention has been given to exploring them together. Metacognitive experiences not only affect metacognitive knowledge by supplementing, revising or deleting knowledge, but also by activating strategy-use for L2 writers (Lee and Mak, 2018; Teng, 2020; see also Papaleontious-Louca, 2008). Metacognitive experiences lead learners to revise original goals and establish new goals that enable progress in their learning process.

Considering the role of metacognitive experiences in the learning process, previous studies have examined metacognitive experiences in specific domains, such as mathematics (e.g., Akama and Yamauchi, 2004) and reading (e.g., Zhang, 2002, 2010). Investigating metacognitive experiences in specific domains provides new perspectives for L2 writing research. However, there is a paucity of research into metacognitive experiences in L2 writing, perhaps surprisingly, given that metacognitive experiences play an important role in language learning. To date, to the best of our knowledge, Wu's (2006) research, mainly concentrated on the affective experiences of metacognitive experiences, was the only study exclusively exploring metacognitive experiences in EFL writing. In addition, little research has addressed the development of instruments for measuring students' metacognitive experiences in the field of L2 writing research, particularly for EFL learners who are most often exposed to English in classroom settings. To fill these lacunae, this study, situated in an EFL learning context, investigates the nature of students' metacognitive experiences in EFL writing through developing and validating a self-report questionnaire, the *EFL Learners' Writing Metacognitive Experiences Questionnaire (EFLLWMEQ)*.

## Review of Literature

### Nature of Metacognitive Experiences

Researchers have defined metacognitive experiences from different perspectives. Metacognitive experiences refer to what an individual goes through during the cognitive process, that is, the individual's “online” metacognitive knowledge, ideas, beliefs, feelings, goals, and judgments (Efklides, 2001). Flavell (1979) defined metacognitive experiences as any kind of affective and cognitive experience but did not elaborate on the subcategories of metacognitive experiences. Efklides (2001) developed Flavell (1979) work by teasing out various aspects of metacognitive experiences in the psychological domain. Metacognitive experiences comprise metacognitive feelings, metacognitive judgments/estimates, and online task-specific knowledge (Efklides, 2001), a composition which is relevant to monitoring and regulating the cognitive process. As Tarricone (2011) states, metacognitive experiences are conscious cognitive and affective experiences, including “awareness, unexpected awareness, thoughts, intuitions, perceptions, feelings and self-judgements of oneself as a cognisor during problem-solving and task completion” (p. 130). Metacognitive experiences are online, that is, occurring during the cognitive process, and specific, occurring in the interface between particular individuals and specific tasks. The adjective “online” distinguishes metacognitive knowledge and strategies that occur spontaneously within metacognitive experiences from the metacognitive knowledge and strategies that make up the solid foundation of learning. The difference between metacognitive knowledge and online metacognitive knowledge includes the fact that metacognitive knowledge is sustained within long term memory whereas online knowledge occurs within working memory.

### Taxonomy of Metacognitive Experiences

There is a growing understanding of the taxonomy of metacognitive experiences in the field of educational psychology. Following Flavell (1979) definition of metacognitive experiences, Efklides (2002a,b) developed a framework of metacognitive experiences encompassing ideas, feelings, judgments, and online metacognitive knowledge in problem-solving process. Metacognitive experiences have personal characteristics associated with individuals' feelings; such metacognitive feelings are the products of nonanalytic and non-conscious inferential processes (Efklides, 2001), that is, they occur spontaneously. Metacognitive feelings can be categorized into the *feeling of difficulty* that arises when the task seems too hard (Efklides et al., 1999); the *feeling of familiarity* when recognizing a previous occurrence of a stimulus and fluency of processing (Nelson, 1996); the *feeling of confidence* that results when individuals reach the answer (Nelson, 1984), the *feeling of satisfaction* when the quality of an answer evidently meets the criteria and standards (Efklides, 2002a), and the *feeling of knowing* and its related tip-of-tongue level of fluency. Those kinds of feelings affect learners' self-regulation decisions. Metacognition regulates cognition through both the cognitive regulatory loop and the affective loop (Efklides, 2009): both conscious thought and spontaneous emotion affects thinking. In this study, the connotations of *emotions* and *feelings* are interchangeable.

It has been established, too, that metacognitive experiences also entail metacognitive judgments/estimates of cognition. Metacognitive judgments/estimates are categorized into *judgment of learning, estimate of solution correctness* (focusing on the quality of the answer), *estimate of time needed or expended, estimate of effort expenditure*, and *episodic memory judgments* (source memory information) (Efklides, 2001). Metacognitive judgments can be products of nonanalytic judgments (Koriat, 2000), occuring instantly without stages of analysis. In addition, metacognitive judgments are linked with metacognitive feelings. Estimates of time and effort for problem-solving processes are often associated with *feeling of difficulty* (Efklides, 2002b; Efklides, 2008). Recognition of correctness leads to a learner's feelings of confidence and satisfaction, and these feelings enable them to monitor the outcomes of problem-solving and thus learn effectively (see also Efklides, 2006). Metacognitive judgments are related to learners' self-monitoring of their own cognition and experiences.

Online task-specific metacognitive knowledge relates to the spontaneous awareness of task-related characteristics and knowledge about both task and strategies in real time. It is a manifestation of the cognitive and analytic processes that individuals need to accomplish a task (Efklides, 2006). Furthermore, online task-specific knowledge also involves episodic memory, so learners draw on past personal experiences when dealing with tasks.

Informed by Efklides (2002a,b) framework of metacognitive experiences in the field of educational psychology, the current study envisions EFL writing metacognitive experiences as a multidimensional construct given that writing is an intricate and recursive process. EFL writing metacognitive experiences subsume (a) metacognitive judgments/estimates; (b) metacognitive feelings; (c) online metacognitive knowledge; and (d) online metacognitive strategies. The first three dimensions of metacognitive experiences are aligned with Efklides (2002a,b) framework in the field of educational psychology. Considering the intricacy of L2 writing, this study additionally proposed the fourth dimension, online metacognitive strategies, referring to metacognitive strategies that students use spontaneously to regulate the writing process in real time. In real time, or “online,” metacognitive experiences can activate metacognitive strategies that control behaviors in the writing process. The proposed framework captures the detailed components of students' metacognitive experiences in the process of writing.

### Research on Metacognitive Experiences in Teaching and Learning

The past few decades have witnessed the investigation of metacognitive experiences from theoretical and empirical perspectives (e.g., Papaleontious-Louca, 2008; Norman and Furnes, 2016). Researchers have concentrated on theoretical analysis, exploring classifications of metacognitive experiences (e.g., Efklides and Vauras, 1999; Efklides, 2001). Efklides (2002a), for example, incorporated metacognitive experiences including metacognitive feelings and judgments/estimates into educational psychology research.

Another strand of research has investigated metacognitive experiences within subject-specific classroom settings. For example, Zhang (2002) observed metacognitive experiences while investigating 160 EFL learners' metacognitive awareness of strategy use in reading. Zhang (2002) used a 36-item Metacognitive Awareness Questionnaire, whose findings revealed that EFL learners' confidence, effectiveness, repair strategy, and perceived difficulty in completing reading tasks were pertinent to performance. Regarding affective experiences, Yu (2020) examined the dimension of enjoyment in EFL learning classroom. The findings revealed that enjoyment of EFL learning had a positive effect on English achievement.

Interest in the significance of metacognitive experiences in teaching and learning led to an interest in questionnaire development. Efklides (2002a) began the process by developing a sematic scale questionnaire exclusively measuring metacognitive experiences, involving prospective and retrospective experiences (i.e., before and after the cognitive process) in the field of educational psychology research. Drawing on Efklides (2002a) metacognitive experiences questionnaire, some researchers have undertaken considerable exploration of metacognitive experiences, and by doing so, achieved an in-depth understanding of metacognitive experiences in the learning process (e.g., Akama, 2007). For example, Akama and Yamauchi (2004) employed Efklides (2002a) metacognitive experiences questionnaire to investigate learners' metacognitive experiences in completing mathematic tasks. They found that there were significantly different metacognitive experiences between successful and unsuccessful learners. Successful learners reported higher feelings of satisfaction, confidence, knowing, and estimates of solution correctness, compared with unsuccessful learners. Further research on metacognitive experiences investigated different teaching contexts. Efklides and Vlachopoulos (2012) also assessed metacognitive experiences in mathematics, and they posited that feeling of difficulty affected the organization of learners' metacognitive knowledge. In the multimedia learning context, Norman and Furnes (2016) broadly examined metacognitive experiences involving predictions of performance, judgments of learning, and confidence ratings, and they found that online learning impeded metacognition compared to in class learning. Recently, Davari et al. (2020) reported on an investigation with 748 Iranian EFL learner. They found that L2 emotions were not a binary structure (i.e., positive and negative emotions), but the results of statistical analysis showed an eight-factor structure of L2 emotions.

Moreover, researchers have been increasingly aware that metacognitive experiences play a crucial role in L2 writing, as experiences interplay with metacognitive knowledge and metacognitive strategies. Kasper (1997) surfaced L2 the significance of students' positive and negative experiences when they wrote an autobiographical passage. Wu (2006) investigated metacognitive experiences through an open-ended questionnaire and journal writing by itemizing the positive and negative metacognitive experiences in EFL writing. Dong and Zhan (2019) examined 56 undergraduates' EFL writing experiences throughout metacognitive instruction. Cognitive and affective experiences, including positive and negative feelings, were found to relate to learning outcomes. Unfortunately, they did not intensively investigate learners' metacognitive experiences in learning to write in EFL. Although an array of theoretical and empirical studies has shown that metacognitive experiences affect learners' performance, there is a paucity of research into metacognitive experiences in L2 writing. Empirical research on the relationship between metacognitive experiences and L2 writing is still cursory, and yet, such research is likely to be of help to both L2 writing instructors and researchers.

### Research on Metacognition in L2 Writing

Research on metacognition in L2 writing has mainly involved three dimensions, namely, metacognitive knowledge, metacognitive experiences, and metacognitive strategies (e.g., Wu, 2006; Karlen, 2017; Zhang and Qin, 2018). Researchers have considered that L2 writing problems may emerge due to a lack of one or more of the metacognitive components related to the nature of the cognitive activity (Negretti and McGrath, 2018; Teng, 2020). Given the interactive relationship between metacognitive experiences, metacognitive knowledge, and metacognitive strategies, this section specifically focuses on metacognitive knowledge and metacognitive strategies in L2 writing.

With regard to metacognitive knowledge in L2 writing, researchers have focused on investigating the subcategories and the influence of metacognitive knowledge (e.g., Victori, 1999; Ruan, 2014). For instance, Kasper (1997) conducted an in-depth study using a questionnaire to assess metacognitive knowledge of participants who were writing autobiographies. This allowed her to examine the role of metacognitive knowledge (e.g., person, task, and strategy knowledge) in L2 writing performance. Results revealed that learners' strategy knowledge was related to their writing performance, while person and task knowledge did not significantly affect the performance of learners with high proficiency level. Ruan (2014) adopted an exploratory study using small group interviews with 51 English-major students to describe Chinese EFL learners' metacognitive awareness in EFL writing, locating details about students' strategy awareness of planning, generating text and revising strategies to create a model for EFL writers to follow. Teng (2020) study found there was a positive relationship between EFL learners' metacognitive knowledge and regulation between writing performance.

Metacognitive strategies could be considered as central to effective foreign language writing performance (Victori, 1999; De Silva and Graham, 2015; Teng and Zhang, 2020). Successful language learners need to deploy various self-regulatory processes, for instance, activating knowledge, and monitoring and regulating their learning process metacognitively (Azevedo, 2009). Language education researchers have acknowledged that the role of metacognitive strategies is critical in developing foreign language writing skills (Bui and Kong, 2019). Using a questionnaire approach, Bai et al. (2014) conducted a study into the relationship between writing strategies and English proficiency based on O'Malley et al. (1990) framework of writing strategies. Students' use of planning, text-generating, revising, monitoring and evaluating, and resourcing strategies were found to be significantly correlated with their writing performance.

Unsurprisingly, given the consistent evidence of relationships between metacognitive components and language proficiency, instructors and researchers have added metacognitive factors in writing instruction to improve learners' writing ability s to develop students' writing ability (e.g., Yeh, 2015; Negretti, 2017; see also, Yu, 2020). For example, De Silva and Graham (2015) adopted stimulated recall to explore the impact of writing strategy instruction on learners with high and low attainment. They found that all learners benefitted from strategy instruction. Negretti and McGrath (2018) found students in the writing class with metacognitive knowledge scaffolds could use genre-based knowledge in their writing. It seems clear that students who know more about metacognitive strategies and how to use them learn and perform better than those with less metacognitive knowledge and strategy (Winne and Hadwin, 1998; Zhang, 2008; Zhang et al., 2016; Zhang and Zhang, 2019).

Taken together, prior research on metacognition in L2 writing provides strong evidence that metacognitive factors contribute to better writing performance. Despite some consideration of metacognition in L2 writing, metacognition has not been sufficiently investigated in the context of EFL writing, that is, the context wherein English writing is learned in a situation where English is rarely used. Arguably, this context provides one of the most challenging learning settings, which cannot be overlooked due to the sheer number of English learners involved in EFL learning contexts. In addition, research on *metacognitive experiences* in L2 writing has not gained much attention given the contribution of metacognitive experiences in the cognitive process, with EFL writing being given little attention. This study is premised on the need to further unravel the nature of EFL student writers' metacognitive experiences in order to assist teachers and learners aiming to improve writing performance.

### Measuring Metacognitive Factors in L2 Writing

Among assessment methods, a questionnaire is a commonly employed instrument for evaluating metacognition (Wirth and Leutner, 2008). The self-report questionnaire is prominently used to gather holistic and comprehensive information about metacognitive factors in foreign language writing. For example, the use of questionnaires provides holistic and large-scale information about learners' metacognitive activity (e.g., Karlen, 2017; Zhang and Qin, 2018; see also Chen et al., 2021). Questionnaires can stimulate and solicit individual perceptions and interpretations of language learners' own learning experiences, gathering data that can provide explanations for their behaviors (Dörnyei, 2011; Iwaniec, 2020).

Metacognitive knowledge and metacognitive strategies are two important constructs for investigating foreign language writing *via* questionnaires. Karlen (2017) assessed metacognitive strategy knowledge for planning, monitoring and revising in academic writing. Results showed satisfactory psychometric properties of his questionnaire. Other researchers also developed new survey instruments for assessing metacognitive strategies in foreign language settings. Zhang and Qin (2018) developed a 23-item instrument for assessing EFL writers' metacognitive strategies in multimedia environments. They found that Chinese EFL learners' writing strategies could be divided into three types, including metacognitive planning, metacognitive monitoring and metacognitive evaluating. Escorcia and Gimenes (2020) developed the Metacognitive Components of Planning Writing Self-Inventory to measure language learners' metacognitive knowledge and self-regulation strategies in writing. Their analysis pointed to three factors: Metacognitive conditional knowledge, covert self-regulation, and environmental self-regulation. So far, available studies have given insight into the metacognitive knowledge and metacognitive strategies language learners use in L2 contexts.

Yet, despite interest in metacognitive knowledge and strategies, questionnaires to capture the nature of metacognitive experiences has not gained much attention. Efklides (2002a) made an initial move, constructing a semantic differential scale questionnaire for assessing individuals' metacognitive experiences in the cognitive process. The questionnaire included two sections, namely, prospective reports and retrospective reports. However, Efklides (2002a) instrument was initially developed for psychological research and is difficult to apply directly to the field of L2 writing research. To date, no models or scales have been exclusively developed to assess metacognitive experiences in EFL writing. The research gaps prompted this study's development of an instrument for investigating students' metacognitive experiences in EFL writing.

## Methods

This study was designed to investigate the multifaceted nature of EFL writers' metacognitive experiences through validating a new questionnaire in an academic EFL learning context. This is because questionnaires are the most promising method for providing insight for the generalization of EFL learners' writing metacognitive experiences. Framed within an adapted framework of metacognitive experiences, the EFL Learners' Writing Metacognitive Experiences Questionnaire (*EFLLWMEQ*) was developed and validated through exploratory factor analysis and confirmatory factor analysis. This study aimed to address the following two research questions:

1. What factorial structure best represents the dimensions of EFL writing metacognitive experiences?
2. If there is an acceptable model fit, how do the dimensions of EFL writing metacognitive experiences correlate with EFL students' writing performance?

### Participants

A total number of 880 students were recruited out of around 10,000 second-year undergraduates through convenience sampling from a national university in Northeast China. Two groups of second-year undergraduates volunteered to participate. Their ages were between 18 and 22 (*M* = 19.69, *SD* = 0.72). On average, these EFL learners had 11.53 (*SD* = 2.11) years of English learning experiences, and Chinese is their mother tongue. They had no study abroad experiences. Even though participants were recruited from different disciplines, their professional majors did not affect their metacognitive experiences of EFL writing as they experienced the same English writing syllabus at a national university. All the participants had enrolled in an English writing course in their second year of undergraduate study. The writing course was designed to improve writing performance and prepare students for the national College English Test (CET). These participants were selected because, as stated by the university, they are permitted to take CET-Band 4 in their second-year study. As such, they had the motivation to participate in this study given that they were under pressure to pass CET-Band 4.

For initial validation of the *EFLLWMEQ*, 340 undergraduates from six faculties at the university were invited to participate. Convenience sampling was used, with participants selected from the Faculties of Earth Science (*N* = 96, 29.4%), Engineering (*N* = 33, 9.0%), Information Science (*N* = 34, 9.4%), Medical Science (*N* = 69, 20.6%), Science (*N* = 55, 16.1%), and Social Science (*N* = 53, 15.5%). Of these participants, 57.7% were males and 42.3% were females. For cross-validation, the second group of participants was 540 second-year undergraduates (66% male, 34% female) from four faculties (Engineering, *N* = 107, 19.8%; Humanities, *N* = 79, 14.6%; Information Science, *N* = 187, 34.6%; Science, *N* = 167, 30.9%).

### Instruments

#### Development of the EFLLWMEQ

Given that there were no existing questionnaires for exclusively measuring EFL writers' metacognitive experiences, we developed the *EFLLWMEQ* through multiple resources: existing literature on metacognitive experiences (e.g., Efklides, 2002a,b; 2009), established questionnaires for assessing metacognition (e.g., Zhang and Qin, 2018), and semi-structured interviews (e.g., Ruan, 2014). We extensively reviewed research on the measurement of metacognition in EFL writing (e.g., Zhang and Qin, 2018), given the overlapping nature of metacognitive knowledge, experience and strategy. Twelve second-year undergraduates from different faculties at one university were invited to participate in the semi-structured interviews for item generation of the *EFLLWMEQ*. Because metacognitive experiences are intrapersonal, undergraduate learners with authentic experiences were chosen to give advice rather than instructors or experts, in alignment with Dörnyei (2011) suggestion that targeted participants in the item-generating process adds to the credibility and quality of questionnaire items. Informed by Efklides (2002a,b) theoretical rationale of metacognitive experiences in the field of psychological research, we proposed a modified multidimensional model of EFL students' writing metacognitive experiences, including metacognitive feelings, metacognitive estimates/judgments, online metacognitive knowledge, and online metacognitive strategies.

Following Dörnyei (2011) guidance on questionnaire development, 28 items were included in the *EFLLWMEQ* under two sections, namely, participants' demographic information, and their metacognitive experiences in EFL writing. A 6-point Likert scale was adopted for measuring EFL writing metacognitive experiences. The reason for choosing a 6-point Likert scale was due to the Chinese EFL learning context. As stated by Cohen et al. (2018), “there is a tendency for participants to opt for the mid-point of a 5-point or 7-point scale. This is notably an issue in East Asian respondents, where the ‘doctrine of the mean’ is advocated in Confucian culture” (p. 327). Adopting 6-point Likert scale was to prevent students from selecting the midpoint. Questionnaire items were scored with numbers from 1 to 6 for responses, ranging from “strongly disagree” to “strongly agree”.

Initial piloting was carried out to check the content and face validity of the instrument. Once a preliminary draft of the metacognitive experiences instrument was completed (drawing on the literature review depicted before), two experts in applied linguistics and educational research scrutinized the initial item pools as a means of ensuring the validity (Petrić and Czárl, 2003). After that, two focus group interviews were conducted with 10 second-year undergraduates and 10 EFL writing instructors to assess the clarity and readability of the *EFLLWMEQ*. As representatives of those who would be using the questionnaire, the undergraduates were invaluable. The EFL writing instructors provided comprehensive feedback on our questionnaire based on their professional expertise. English language was used for the questionnaires as the undergraduates had a reasonably good command of English vocabulary. They have gone through College Entrance Exam and averagely learned English for more than 11 years. Also, questionnaire translation from English to Chinese might change the original meaning of the items, and translation back into English could also introduce slippage. The choice to use English meant that the wording of questionnaire items needed to be both accurate and simple. Initial piloting led to revising two double-barreled items and deleting four unnecessary items. The *EFLLWMEQ* with 24 items was generated with no initial problems.

#### Writing Test

In this study, participants who enrolled in the writing course were invited to complete a writing task of at least 150 words on a given topic in 30 min in the classroom setting. The composition in this study was an argumentative writing task that was selected and modified from the CET-Band 4. An argumentative writing task is a typical genre that university students encounter in English proficiency tests, such as CET and the International English Language Testing System (IELTS). It is an effective approach to evaluate students' writing performance based on their linguistic competence, critical thinking, and articulation of ideas (Hirose, 2003). The validity of CET has been widely recognized, and 30 min is allowed in the standard CET. Therefore, we adopted and revised one writing task from CET-4 as the writing prompt for this study (see Appendix B). The writing topic selected for this study was general, culturally inoffensive, and familiar to undergraduate students' experiences.

The assessment of students' writing performance was in accordance with Jacobs et al. (1981) ESL Composition Profile. The rubrics of this profile evaluated five aspects of writing performance, namely, content (30%), organization (20%), language use (25%), vocabulary (20%), and mechanics (5%). Each subcategory had four rating levels. Two experienced EFL writing instructors were invited to mark students' compositions. A training session was conducted, and each rater assessed 100 writing compositions, i.e., around 20% of the samples, and compared their scores. They discussed any discrepancies with reference to the composition profile. The inter-rater reliability was *r* = 0.83, *p* < 0.001, indicating acceptable reliability. After the training session, the two raters assessed the remaining writing compositions separately.

### Data Collection

In the initial validation of the *EFLLWMEQ*, a total of 340 self-report questionnaires were distributed to students after the first session of the writing course at the beginning of the semester. On average, students spent approximately 15 min completing the *EFLLWMEQ*. At the end of the semester, another sample of 540 participants enrolled in the writing course was first required to finish an argumentative writing task. After that, in the classroom setting, students completed the refined version of the *EFLLWMEQ* that elicited their authentic context-based metacognitive experiences. To enhance the reliability and validity of this study, in-class tests and subsequent time constraints were designed to control complicating factors. For instance, students were not able to look up the dictionary or search the information online. Students were required to finish the writing task within 30 min.

### Data Analysis

#### Exploratory Factor Analysis

The exploratory factor analysis (EFA) was adopted as a data reduction technique to summarize variables into a small set of factors (Allen et al., 2014). We used EFA to investigate the dimensions underlying the EFLLWMEQ. Before conducting EFA, we eliminated invalid data, including mischief answers and missing values from the database. The assumptions of linearity, singularity and homogeneity of the sample were thoroughly checked, and no outliers were detected. After data screening and cleaning, the sample size of 310 cases was then subjected to EFA. The sample size of this study also met the desired case-and-variable ratio as there were at least five cases for each of the variables (Field, 2018).

The 24 items of the *EFLLWMEQ* were subjected to principal axis factoring (PAF) analysis with an oblique rotation (direct oblimin) using IBM SPSS Version 26.0. This is because we assumed that the items might have shared sources of error, and the factors of the measured structure were interrelated (Tabachnick and Fidell, 2007; Pallant, 2016). To decide the number of retaining factors, the results of Kaiser (1960) eigenvalues-greater-than-one (K1) rule, the scree test and the parallel analysis were adopted (Pallant, 2016; Field, 2018). The cut-off value for a significant factor loading was set at 0.32 (Tabachnick and Fidell, 2007), as the first round of the sample size of this study was over 300.

#### Confirmatory Factor Analysis

The second round of data was subjected to confirmatory factor analysis (CFA). CFA aimed to cross-validate the structures generated in EFA to assess the construct validity and discriminant validity of the newly-developed questionnaire (Awang, 2012). IBM SPSS AMOS Version 26.0 was used to examine the factorial structure underlying the *EFLLWMEQ*. Maximum likelihood (ML) estimation, a technique for CFA, was employed to evaluate the model parameters and model fit indices (Tabachnick and Fidell, 2007). Prior to conducting CFA, all the assumptions of normality, linearity and homogeneity of the collected data for multivariate analysis were checked. Given the sensitivity of CFA to outliers and missing values, we also thoroughly scrutinized the data. After data screening and cleaning, the final sample size was 513 that met the desired cases-to-variables ratio (5:1) for conducting CFA (Field, 2018).

Data collected from the revised version of the *EFLLWMEQ* were analyzed through several omnibus fit statistical analyses to assess the goodness-of-fit of the hypothesized model. Although there are no golden rules for evaluating model fit, the chi-square statistic (χ^2^) and its degrees of freedom (*df* ) and *p*-value are the fundamental statistics when researchers report CFA results (Kline, 2015). Nevertheless, chi-square values are sensitive to sample size; for example, chi-square values may yield a statistically significant result with a large sample size (Hooper et al., 2008). As such, we also consulted three absolute fit indices that were recommended by Hair et al. (2010), namely, the value of the ratio of χ^2^ divided by its degree of freedom (χ^2^/*df* ); the standardized root mean square residual (SRMR); and the root mean square error of approximation (RMSEA) with its corresponding 90% confidence interval (Steiger, 1990). We also considered the fact that the value of χ^2^/*df* less than 3.0 with a non-significant *p*-value indicates the best model fit to accept the null hypothesis. There is no difference between implied variances and covariances of a model, and the observed variance and its covariance (Marsh et al., 1988). Hu and Bentler (1999) propose that the recommended values of RMSEA are ≤ 0.05, indicating a generally acceptable model fit, and the recommended values for SRMR are <0.05. Two incremental fit indices, the comparative fit index (CFI) and the Tucker and Lewis index (TLI), were taken into consideration (Tucker and Lewis, 1973; Bentler, 1990). Models with threshold values of CFI and TLI are equal to or >0.90, indicating acceptable model fit (Hu and Bentler, 1999).

Although the model fit indices can describe the model specification, those indices are affected by model parsimony or degrees of freedom (Byrne, 2016). As the value of Gamma Hat is not affected by sample size (Fan and Sivo, 2007), Gamma Hat was reported in this study as a powerful index. The recommended value of Gamma Hat is >0.90, which indicates an acceptable model fit. Given that there is no consensus for the threshold values of model fit indices, these indices are guidance rather than stringent rules for evaluating model fit (Hooper et al., 2008). Table 1 presents the threshold values of goodness-of-fit indices.

**Table 1:** Benchmarks of Goodness-of-Fit indices.

**Indices**	**χ 2/*df***	**TLI**	**CFI**	**Gamma Hat**	**SRMR**	**RMSEA**
Acceptable value	≤3.0	≥0.90	≥0.90	≥0.90	≤0.08	≤0.06

#### Relationship Between EFLLWMEQ Scores and Writing Test Scores

The relationship between the proposed model and writing performance was tested through Pearson product-moment correlation analysis. The correlation analysis was performed to investigate the relationship between the identified factors of EFL writing metacognitive experiences and EFL learners' writing test scores. Effect size was reported to show the relationship between EFL writing metacognitive experiences and EFL writing performance.

## Results

### Results of EFA

Descriptive analysis of the *EFLLWMEQ* revealed that the mean scores of 24 items ranged from 3.12 to 4.52, with the standard deviations from 1.06 to 1.32. The values for skewness and kurtosis of 24 items were within the critical points of |3.0| and |8.0| respectively (Kline, 2015), indicating the normal distribution of data for EFA.

The KMO measure was 0.909 (“marvelous” according to Hutchson and Hutcheson and Sofroniou (1999), p. 78), suggesting the adequacy of the sample size for EFA. The inspection of Bartlett's test of sphericity (χ^2^ = 2956.283, *df* = 276, *p* < 0.001) and correlation matrix of questionnaire items (the presence of many coefficients of 0.32 and above) indicated that the *EFLLWMEQ* was suitable for EFA. The initial commonalities of all the *EFLLWMEQ* items ranged from 0.286 to 0.607. Item 1 was removed as its factor loading was 0.20, which was less than the threshold value (Allen et al., 2014). Items with factor loadings >0.32 and no crossing-loadings were retained for further statistical analysis (Pallant, 2016; Field, 2018). After several iterative rounds of EFA, a four-factor model with 21 items (items 1, 3, and 11 were excluded) was generated, which accounted for 57.12% of the total variance on the earlier version of the *EFLLWMEQ*. PFA analysis of the retained 21 items confirmed a four-factor model, explaining 57.12% of the total variance.

The items that loaded onto each of the four factors were analyzed thematically for the purpose of identifying a potential construct that the *EFLLWMEQ* represented. Through the examination of items clustering, four factors were identified and labeled as: Factor 1, *Metacognitive Estimates of EFL Writing (MEEFLW)*, consisted of seven items including items 18, 19, 20, 21, 22, 23, and 24, accounting for 34.91% variance; Factor 2 was named as *Metacognitive Feelings of EFL Writing (MFEFLW)*, involving items 2, 4, 5, 6, 7, 16, and 17, accounting for 10.61% variance; Factor 3, *Online Metacognitive Knowledge of EFL Writing (OMKEFLW)* had three items (items 13, 14, and 15), accounting for 6.52% variance; Factor 4, *Online Metacognitive Strategies of EFL Writing (OMSEFLW)*, including items 8, 9, 10, and 11, accounting for 5.09% variance.

Reliability analysis was conducted to check the internal consistency of the *EFLLWMEQ*. The Cronbach's alpha coefficient was employed to reduce the subscale of a questionnaire, including multiple factors (Field, 2018). The values of Cronbach's alpha coefficient for the subscales of the *EFLLWMEQ* were high. Cronbach's alpha coefficient for the four factors ranged from 0.700 for Factor 3 to 0.852 for Factor 2. The internal consistency for the four-factor scale was much higher than the benchmark value of 0.60 that DeVellis (2012) proposed, which suggested robust reliability of the *EFLLWMEQ*. Table 2 shows the factor loadings of EFA results and internal reliabilities of the *EFLLWMEQ* subscales.

**Table 2:** Factor loadings and reliability of the *EFLLWMEQ* (*N* = 310).

**Factor**	**Item**	**Factor Loading**				
		**1**	**2**	**3**	**4**	**α**
Factor 1	Item22	0.632				0.852
	Item23	0.541				
	Item20	0.534				
	Item21	0.529				
	Item18	0.487				
	Item24	0.459				
	Item19	0.423				
Factor 2	Item 7		0.759			0.807
	Item16		0.687			
	Item 5		0.626			
	Item17		0.543			
	Item 6		0.514			
	Item 2		0.506			
	Item 4		0.477			
Factor 3	Item13			0.633		0.700
	Item14			0.592		
	Item15			0.419		
Factor 4	Item11				0.777	0.827
	Item10				0.670	
	Item 8				0.619	
	Item 9				0.508	

*Items with a factor loading of 0.32 or greater are included; α = Cronbach's alpha*.

### Cross-Validation of the EFLLWMEQ

Multivariate normality was checked through Mardia's coefficient and multivariate kurtosis critical ratios. The value of Mardia's coefficient for examining multivariate normality was 59.856, which was much less than the recommended value 288 calculated from the formula *p* (*p* + 2), where *p* is the total number of observed indicators (Raykov and Marcoulides, 2008). Multivariate kurtosis critical ratios were also less than the cut-off value of five (Byrne, 2016), also indicating multivariate normality of the data. No outliers were detected using Mahalanobis distance. All assumptions for multivariate analysis revealed that the data were sufficient to conduct CFA.

#### Results of CFA

A four-factor correlated model with 21 items was proposed in EFA, where each indicator was constrained to load only on the first-order factor it was designed to measure. Factor covariance's were free to be estimated, and error terms associated with each indicator were uncorrelated. Initial CFA results for the four-factor model with 21 items were not fully satisfactory (χ^2^ = 559.840; *df* = 183; *p* < 0.001; χ^2^/*df* = 3.059; TLI = 0.859; CFI = 0.877; RMSEA = 0.063 [0.057, 0.069]; SRMR = 0.0623; Gamma hat = 0.94). In reviewing modification indices, we made attempts to improve the model fit of the four-factor correlated model. In the end, Items 4, 6, 17, 18 and 19 were removed from the four-factor model as they had strong error covariance and standardized residual covariances with other items. Thus, the results of CFA revealed a four-factor correlated model with 16 items.

**Figure 1:**
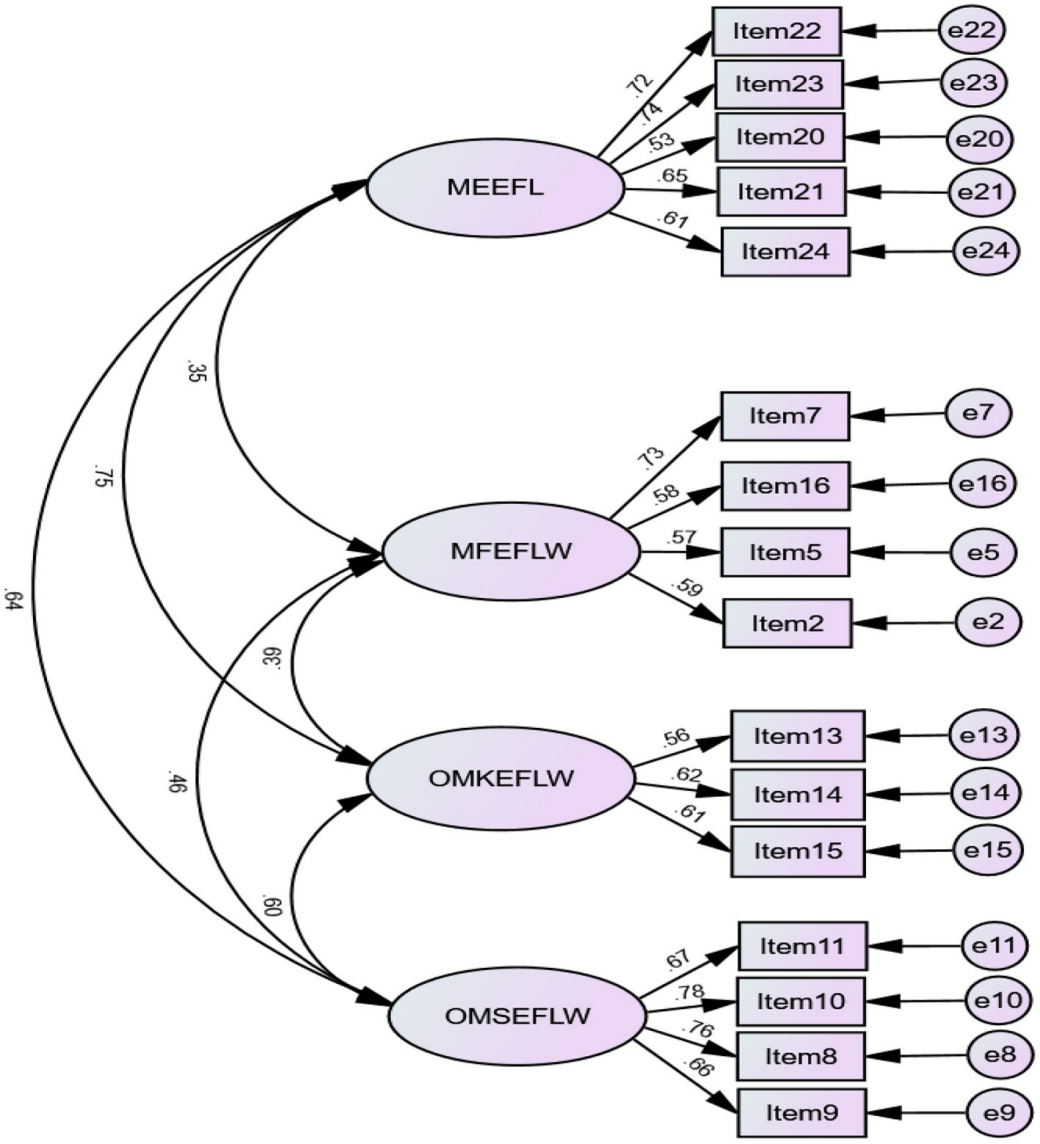
A Four-Factor Model of EFL Writing Metacognitive Experiences (*N* = 513). MEEFLW, Metacognitive Estimates of EFL Writing; MFEFLW, Metacognitive Feelings of EFL Writing; OMKEFLW, Online Metacognitive Knowledge of EFL Writing; OMSEFLW, Online Metacognitive Strategies of EFL Writing.

With reference to convergent validity of the *EFLLWMEQ*, all the 16 items were statistically significant (*p* < 0.001) with standardized estimates loadings higher than 0.50, showing an acceptable effect size (Hair et al., 2010). The threshold value of standardized estimates loading is 0.50 for newly-developed questionnaires (Awang, 2012). The CFA results of the revised model with 16 items indicated an acceptable model fit with χ^2^ = 279.672; *df* = 98; *p* < 0.001; χ^2^/*df* = 2.854; TLI = 0.908; CFI = 0.925; RMSEA = 0.060 [.052,0.069]; SRMR = 0.049; Gamma hat = 0.960. The number of indicators for each factor was greater than two. Given that no remaining modification was theoretically justifiable, no *post-hoc* modifications were conducted. Figure 1 shows the four-factor correlated model of the *EFLLWMEQ*.

#### Model Comparisons

Model comparison is a highly recommended procedure to evaluate the construct validity of factorial structure for scale development (Hair et al., 2010; Kline, 2015; Teng and Zhang, 2016). We proposed three comparison models by using a succession of CFA for developing a theoretical justified model, including a one-factor unidimensional model with all 16 items loading on a single latent factor (Model 1); a four-factor uncorrelated model (Model 2); and a four-factor correlated model with the four factors related to one another (Model 3). So, the proposed correlated model (Model 3) was compared with the other two models (i.e., Model 1 and 2) to test its construct validity (see Table 3). We consulted the chi-square difference test with the corresponding change of the degrees of freedom to evaluate the significant differences among the three models (Kline, 2015). We fitted the data into Model 1 (χ^2^ = 796.979; *df* = 104; *p* < 0.001; χ^2^/*df* = 7.663; TLI = 0.670; CFI = 0.714; RMSEA = 0.114; SRMR = 0.877; Gamma hat = 0.860); Model 2 (χ^2^ = 704.254; *df* = 104; *p* < 0.001; χ^2^/*df* = 6.772; TLI = 0.714; CFI = 0.752; RMSEA = 0.106; SRMR = 0.205; Gamma hat = 0.870); and Model 3 (χ^2^ = 279.672; *df* = 98; *p* < 0.001; χ^2^/*df* = 2.854; TLI = 0.908; CFI = 0.925; RMSEA = 0.060; SRMR = 0.049; Gamma hat = 0.96) respectively. Model fit indices of Model 3 were statistically better than Model 1 ($\chi_{diff}^{2}=517.307$,*df*_*diff*_ = 6, *p* < 0.001) and Model 2 ($\chi_{diff}^{2}=517.307$,*df*_*diff*_ = 6, *p* < 0.001). CFA results of Model 3 (the four-factor correlated model) reached an acceptable model fit. The 16-item parameter estimates were statistically significant, and standardized estimates loadings on the hypothesized latent variables were greater than the cut-off point 0.50, showing an acceptable effect size (Raykov and Marcoulides, 2008). Therefore, the four-factor correlated model was retained in this study, supporting the construct validity and convergent validity of the *EFLLWMEQ* (see Appendix A for the finalized version).

**Table 3:** Model fit indices for the three models.

**Indices**	**χ 2/*df***	**TLI**	**CFI**	**Gamma Hat**	**SRMR**	**RMSEA**
Model 1	7.663	0.670	0.714	0.860	0.877	0.114
Model 2	6.772	0.714	0.752	0.870	0.205	0.106
Model 3	2.854	0.908	0.925	0.960	0.049	0.060

#### Relationship Between EFL Writing Metacognitive Experiences and Writing Performance

The correlation matrix revealed the four factors of the *EFLLWMEQ* were significantly correlated with moderate degrees, as Table 4 presents. The adequate range of correlations ranged from 0.353 (*MEEFLW* and *MFEFLW*) to 0.753 (*MEEFLW* and *OMKEFLW*), which confirmed the discriminant validity of the *EFLLWMEQ*. These four factors were correlated but were also distinct constructs. Table 4 also shows the correlations between four types of EFL learners' metacognitive experiences and the writing test scores. The writing scores were positively correlated with metacognitive estimates of EFL writing (*r* = 0.118, *p* < 0.05) and online metacognitive knowledge of EFL writing (*r* = 0.100, *p* < 0.05), showing a small effect size. Nevertheless, there were no significant relationships between EFL learners' writing scores and the other two subcategories of EFL writing metacognitive experiences, that is, metacognitive feelings and online metacognitive strategies.

**Table 4:** Correlation matrix for the four-factor model and writing performance.

	**MEEFLW**	**MFEFLW**	**OMKEFLW**	**OMSEFLW**	**Writing Performance**
MEEFLW	1				
MFEFLW	0.353^**^	1			
OMKEFLW	0.753^**^	0.387^**^	1		
OMSEFLW	0.639^**^	0.462^**^	0.600^**^	1	
Writing Performance	0.118^*^	0.078	0.100^*^	0.073	1

*MEEFLW, Metacognitive Estimates of EFL Writing; MFEFLW, Metacognitive Feelings of EFL Writing; OMKEFLW, Online Metacognitive Knowledge of EFL Writing; OMSEFLW, Online Metacognitive Strategies of EFL Writing*;

* *p < 0.05*,

** *p < 0.001*.

## Discussion

This study aimed to further research investigation of EFL writers' metacognitive experiences through validating a questionnaire with psychometric properties and contributing it to the writing research community. Data collected from the *EFLLWMEQ* were subjected to EFA and CFA to ensure validity and reliability (Hair et al., 2010), and results showed a four-factor correlated model of EFL writing metacognitive experiences, including metacognitive estimates, metacognitive feelings, online metacognitive knowledge, and online metacognitive strategies. Cross-validation of the *EFLLWMEQ* provided empirical evidence for the psychometric property of the questionnaire in terms of construct validity, discriminant validity, and internal reliability. The four-factor correlated model indicated that the four factors were distinctive but correlated constructs underlying EFL writing metacognitive experiences. Therefore, scores of the *EFLLWMEQ* with 16 items capture the richness of individual students' EFL writing metacognitive experiences. A higher score reveals that EFL learners have richer metacognitive experiences in the course of learning to write. The positive relationship between students' EFL writing metacognitive experiences and writing performance was supported by the results of correlation analysis.

Factor one, labeled metacognitive estimates of EFL writing, including five items, refers to EFL learners' judgments of their effort (e.g., *I pay attention to vocabulary use in my writing*) and time expenditure in EFL writing. In examining this category of metacognitive experiences, we found that EFL learners tried to estimate their effort expenditure in the process of writing regarding vocabulary use, grammar use, sentence structures, and organization. Moreover, students paid attention to the time needed or expended (e.g., *I check if I finish this writing task on time*). This might be attributed to examination culture in the Chinese EFL learning context, even though process-oriented and genre-oriented writing instruction is advocated in the Chinese EFL learning context (see Zhang and Zhang, 2013; Yeh, 2015). In our study, results showed that metacognitive estimates of EFL writing were significantly correlated with EFL learners' writing scores. Unsurprisingly, in the process of EFL writing, students who concentrate on vocabulary use, language use, and organization would normally complete writing tasks within time as they expect. This finding aligns with some existing studies on the relationship between metacognitive judgments and learning (e.g., Norman and Furnes, 2016; Negretti, 2017). These findings indicate that learners' metacognitive judgments contribute to their learning performance.

Factor two, including four items, was named metacognitive feelings of EFL writing. This category refers to the affective character of metacognitive experiences in EFL writing process. Findings showed that EFL learners produce metacognitive feelings comprising feeling of confidence (e.g., *I feel confident about myself as a writer*) and feeling of satisfaction (e.g., *I am satisfied with my writing*) in the course of fulfilling writing tasks. These metacognitive feelings are nonanalytic, nonconscious, and retrospective, as reiterated by Efklides (2002a,b); Efklides (2006). The results of our study are in line with some existing studies on emotions in the field of L2 learning research (e.g., Kasper, 1997; Zhang, 2002; Wu, 2006; Davari et al., 2020). The findings reveal that emotions such as confidence and enjoyment occurred in EFL writing. Our findings provide insight into EFL writing instruction. Teachers should enrich students' positive feelings and alleviate negative feelings in L2 writing instruction classes. Differing from some previous studies on emotions in L2 learning (e.g., Jin and Zhang, 2021), no significant relationship was found between metacognitive feelings and writing performance. This might be attributed to high-stakes testing culture in the Chinese EFL learning context (Carless, 2011). Students did not pay much attention to feelings in learning to write, perhaps because scores were the focus rather than emotions.

Factor three, named online metacognitive knowledge of EFL writing, with three items, depicts EFL learners' online metacognitive knowledge about writers themselves (e.g., *I learn/write more if I am interested in this writing topic*), task knowledge (e.g., *I ensure the first and last sentences are strong enough to explain my meaning*), and strategy knowledge (e.g., *I use what I have learned from my English courses*). It should be noted that, in our study, metacognitive estimates are nonanalytic, while online metacognitive knowledge is task-specific and analytic in EFL writing process. The results of this study reveal that EFL learners tended to employ their online metacognitive knowledge, including person, task, and strategy knowledge to facilitate their writing performance. This finding also showed a significant positive correlation between online metacognitive knowledge of EFL writing and students' writing scores, endorsing previous research (e.g., Negretti and McGrath, 2018; Teng, 2020). The consistency of findings indicates the interactional nature of metacognitive knowledge in EFL writing process. Metacognitive knowledge is centrally involved in the monitoring and regulation of language learning (Wenden, 1998). As such, students who achieve better writing performance have applied their online metacognitive knowledge involving person, task, and strategy dimensions.

Factor four, online metacognitive strategies of EFL writing, included four items, and refers to EFL learners' online metacognitive strategies while composing a writing task (e.g., *I check my spelling*). In examining these four items, we found that EFL learners tried to adopt multiple metacognitive strategies (i.e., metacognitive monitoring and evaluating), to improve their writing performance. Note that online metacognitive strategies are included in EFL writing metacognitive experiences because, in real time, students orchestrated clusters of metacognitive strategies regarding the writing process (i.e., task-specific metacognitive strategies). Students reported use of metacognitive monitoring and evaluating related to language use, organization, and mechanics of writing. This finding aligns with some prior studies on metacognitive strategies in EFL writing (e.g., Zhang and Qin, 2018; Dong and Zhan, 2019). Nevertheless, EFL learners' online metacognitive strategies did not have a statistically significant correlation with their writing scores in our study, which is not consistent with results of some existing research (e.g., Bai et al., 2014; De Silva and Graham, 2015). A possible explanation for this might be that in our one-off study, students might have applied low calibration of their writing performance, and thus failed to perform as well as they expected. Further research could investigate this discrepancy.

Taken together, our findings confirm that EFL writing metacognitive experiences include both affective and cognitive regulatory loops, which suggest EFL learners who have relatively intense metacognitive experiences tend to perform better in learning to write. The findings of our study differed from Efklides (2001) research findings that the relationship between learners' metacognitive experiences and task performance was not significantly strong. This inconsistency is perhaps due to types of tasks. We adopted an EFL writing task, whereas Efklides (2001) employed mathematic tasks. Results of our study suggest that EFL writing metacognitive experiences involve students' estimate of effort expenditure, estimate of time expended, feeling of confidence, feeling of satisfaction, online metacognitive knowledge (i.e., person, task, strategy knowledge), and online metacognitive strategies (i.e., self-monitoring and self-evaluating) in completing a writing task. Given the overlapping interaction among metacognitive experiences, metacognitive knowledge, and metacognitive strategies, EFL learners' metacognitive experiences also demonstrate their employment of online metacognitive knowledge and orchestration of online metacognitive strategies in the EFL writing process (see also Lee and Mak, 2018). Although we found a weak correlation between EFL metacognitive experiences and writing scores, this study has provided preliminary empirical evidence that the richness of EFL learners' metacognitive experiences probably affects their writing performance. Metacognitive experiences, including both judgments and feelings monitor the outcome of a problem-solving process (Efklides, 2012). Such intense metacognitive experiences help facilitate students' attainment of better EFL writing performance as metacognitive experiences mediate in the interface between EFL writers and writing tasks.

## Conclusion

This study was designed to investigate students' metacognitive experiences in learning to write in EFL through a newly-developed questionnaire (the *EFLLWMEQ*). To the best of our knowledge, this study serves as an initial attempt to exclusively measure EFL learners' writing metacognitive experiences in an EFL learning context. Framed within an adapted framework of metacognitive experiences, data collected from the *EFLLWMEQ* substantiated a four-factor correlated model, namely, metacognitive estimates of EFL writing, metacognitive feelings of EFL writing, online metacognitive knowledge of EFL writing, and online metacognitive strategies of EFL writing. The four-factor model of EFL writing metacognitive experiences indicates that the *EFLLWMEQ* was a reliable diagnostic instrument with good validity and reliability, indicating the construct of metacognitive experiences is multidimensional. One novel finding of this study was that metacognitive estimates and online metacognitive knowledge of EFL writing were significantly correlated with EFL learners' writing performance.

Our findings have some implications. Theoretically, the present study helps enrich the taxonomy of metacognitive experiences in learning to write in EFL. The findings broaden understanding of the nature of EFL writing metacognitive experiences, and the relationship between metacognitive experiences and writing performance in EFL learning contexts. The findings of this study lend support to the transferability of metacognitive experiences framework from educational psychology to EFL writing. Our newly-developed instrument with robust validity and reliability for measuring EFL writing metacognitive experiences makes a methodological contribution to the research community. Given that the *EFLLWMEQ* was validated as a reliable diagnostic instrument, researchers could deploy this questionnaire to investigate EFL writing metacognitive experiences in a similar learning context. EFL writing instructors might want to use the *EFLLWMEQ* to assess students' metacognitive experiences in learning to write in the classroom setting. Likewise, EFL learners could use it to self-diagnose their EFL writing metacognitive experiences, enabling them to have a better understanding of how they can make use of metacognitive experiences. The investigation of EFL writing metacognitive experiences sheds light on the importance of metacognitive experiences for EFL teachers and students. Considering the importance of metacognitive experiences, L2 researchers and practitioners need to pay attention to learners' metacognitive experiences in language learning. With reference to meta cognitively oriented instruction, teachers should not only focus on fostering students' metacognitive knowledge and metacognitive strategies, but also help students develop a rich repertoire of metacognitive experiences to expedite their learning-to-write process and improve their writing performance in L2 learning contexts.

Despite the significant findings, like any other study, ours also suffers limitations that need to be addressed in future studies. Due to the convenience sampling, this study only recruited undergraduates in the Chinese EFL learning context. Therefore, it may not always be suitable to generalize the findings of this study to EFL learners in other learning contexts. In addition, we measured EFL learners' metacognitive experiences after they finished a writing task, but students' metacognitive experiences are dynamic during the writing process. This study did not examine the dynamic nature of the construct, metacognitive experiences. A qualitative study into EFL learners' dynamic change of metacognitive experiences when they learn to write is well in order. Multiple methods for data collection, for example, interviews and reflective journals, are recommended for better understanding of EFL learners' metacognitive experiences in learning to write.

## Data Availability Statement

The original contributions presented in the study are included in the article, further inquiries can be directed to the corresponding author.

## Ethics Statement

The studies involving human participants were reviewed and approved by The University of Auckland Human Ethics Committee. The patients/participants provided their written informed consent to participate in this study.

## Author Contributions

QS conceived of the initial idea, fine-tuned by LZ and SC. QS designed the study, collected and analyzed the data, and drafted of the manuscript. LZ and SC revised and proofread the manuscript. All authors agreed to the final version before LZ got it ready for submission as the corresponding author.

## Conflict of Interest

The authors declare that the research was conducted in the absence of any commercial or financial relationships that could be construed as a potential conflict of interest.

## Publisher's Note

All claims expressed in this article are solely those of the authors and do not necessarily represent those of their affiliated organizations, or those of the publisher, the editors and the reviewers. Any product that may be evaluated in this article, or claim that may be made by its manufacturer, is not guaranteed or endorsed by the publisher.

## References

[B1] Akama K. (2007). Previous task experience in metacognitive experience. Psychol. Rep. 100, 1083–1090. 10.2466/pr0.100.4.1083-1090 17886492

[B2] Akama K. Yamauchi H. (2004). Task performance and metacognitive experiences in problem-solving. Psychol. Rep. 94, 715–722. 10.2466/pr0.94.2.715-722 15154206

[B3] Allen P. Bennett K. Heritage B. (2014). SPSS statistics version 22: a practical guide. 3rd ed. Boston, MA: Cengage Learning.

[B4] Awang Z. (2012). Structural Equation Modeling Using AMOS Graphic. Shah Alam: Universiti Technologi MARA Publication Centre.

[B5] Azevedo R. (2009). Theoretical, conceptual, methodological, and instructional issues in research on metacognition and self-regulated learning: a discussion. Metacognition Learn. 4, 87–95. 10.1007/s11409-009-9035-7

[B6] Bai R. Hu G. Gu P. Y. (2014). The relationship between use of writing strategies and English proficiency in Singapore primary schools. Asia-Pacific Educ. Res. 23, 355–365. 10.1007/s40299-013-0110-0

[B7] Bentler P. M. (1990). Comparative fit indexes in structural models. Psychol. Bull. 107, 238–246. 10.1037/0033-2909.107.2.238 2320703

[B8] Bui G. Kong A. (2019). Metacognitive instruction for peer review Interaction in L2 writing. J. Writ. Res. 11, 357–392. 10.17239/jowr-2019.11.02.05

[B9] Byrne B. M. (2016). Structural Equation Modeling With AMOS. 3rd ed. New York, NY: Routledge.

[B10] Carless D. (2011). From Testing to Productive Learning: implementing Formative Assessment in Confucian-Heritage Settings. New York, NY: Routledge.

[B11] Chen J. Zhang L. J. Wang X. Zhang T. T. (2021). Influences of SRSD revision instruction on English-as-a-foreign-language (EFL) students' self-efficacy for text revision: a mixed-methods study. Front. Psychol. 12, 1–14. 10.3389/fpsyg.2021.670100 34335382 PMC8321093

[B12] Cohen L. Manion L. Morrison K. (2018). Research Methods in Education (8th ed.). New York, NY: Routledge.

[B13] Davari H. Karami H. Nourzadeh S. Iranmehr A. (2020). Examining the validity of the achievement emotions questionnaire for measuring more emotions in the foreign language classroom. J. Multiling. Multicult. Dev. 176:6054. 10.1080/01434632.2020.1766054

[B14] De Silva R. Graham S. (2015). The effects of strategy instruction on writing strategy use for students of different proficiency levels. System 53, 47–59. 10.1016/j.system.2015.06.009

[B15] DeVellis R. F. (2012). Scale Development: Theory and Application. 3rd ed. London: Sage Publications.

[B16] Devine J. (1993). The role of metacognition in second language reading and writing, in Reading in the composition classroom: Second language perspectives, eds. Carson J. G. Leki I. (Boston: Heinle and Heinle), 105–127.

[B17] Dong X. Zhan J. (2019). Impacts of metacognitive instruction on college students' EFL writing metacognitive features: knowledge experiences and strategies. Foreign Lang. China 16, 62–70. 10.1080/02188791.2020.1835606

[B18] Dörnyei Z. (2011). Research Methods in Applied Linguistics: Quantitative, Qualitative, and Mixed Methodologies. Oxford: Oxford University Press.

[B19] Efklides A. (2001). Metacognitive experiences in problem solving: metacognition, motivation, and self-regulation, in Trends and Prospects in Motivation Research, eds Efklides A. Kuhl J. Sorrentino R. M. (New York, NY: Kluwer Academic Publishers), 297–323.

[B20] Efklides A. (2002a). Feelings as subjective evaluations of cognitive processing: how reliable are they? Psychol. J. Hell. Psychol. Soc. 9, 163–182.

[B21] Efklides A. (2002b). The systemic nature of metacognitive experiences: feelings, judgments, and their interrelations., in Metacognition: Process, Function and Use, eds. Chambres P. Izaute M. Marescaux P. J. (New York, NY: Kluwer Academic Publishers), 19–34.

[B22] Efklides A. (2006). Metacognition and affect: what can metacognitive experiences tell us about the learning process? Educ. Res. Rev. 1, 3–14. 10.1016/j.edurev.2005.11.001

[B23] Efklides A. (2009). The role of metacognitive experiences in the learning process. Psicothema 21, 76–82. 19178860

[B24] Efklides A. (2012). Commentary: how readily can findings from basic cognitive psychology research be applied in the classroom. Learn. Instr. 22, 290–295. 10.1016/j.learninstruc.2012.01.001

[B25] Efklides A. Samara A. Petropoulou M. (1999). Feeling of difficulty: an aspect of monitoring that influences control. Eur. J. Psychol. Educ. 14, 461–476. 10.1007/BF03172973

[B26] Efklides A. Vauras M. (1999). Introduction. Eur. J. Psychol. Educ. 14, 455–459. 10.1007/BF03172972

[B27] Efklides A. Vlachopoulos S. P. (2012). Measurement of metacognitive knowledge of self, task, and strategies in mathematics. Eur. J. Psychol. Assess. 28, 227–239. 10.1027/1015-5759/a000145

[B28] Escorcia D. Gimenes M. (2020). Metacognitive components of writing: construction and validation of the metacognitive components of planning writing self-inventory (MCPW-I). Rev. Eur. Psychol. Appl. 70:100515. 10.1016/j.erap.2019.100515

[B29] Fan X. Sivo S. A. (2007). Sensitivity of fit indices to model misspecification and model types. Multivariate Behav. Res. 42, 509–529. 10.1080/00273170701382864

[B30] Field A. (2018). Discovering statistics using IBM SPSS statistics. Thousand Oaks, CA: Sage.

[B31] Fisher L. (2018). ‘Emotion recollected in tranquillity’ blogging for metacognition in language teacher education, in Metacognition in Language Learning and Teaching, eds. Haukås Å. Bjørke C. Dypedahl M. (New York, NY: Routledge) 224–242. 10.4324/9781351049146-12

[B32] Flavell J. H. (1976). Metacognitive aspects of problem solving, in The nature of intelligence, ed. Resnick L. R. (Hillsdale,NJ: Lawrence Elbaum).

[B33] Flavell J. H. (1979). Metacognition and cognitive monitoring: a new area of cognitive-developmental inquiry. Am. Psychol. 34, 906–911. 10.1037/0003-066X.34.10.906

[B34] Garner R. (1987). Metacognition and Reading Comprehension. New York, NY: Ablex Publishing.

[B35] Hacker D. J. Dunlosky J. Graesser A. C. (2009). Handbook of Metacognition in Education. New York, NY: Routledge.

[B36] Hair J. F. Black W. C. Babin B. J. Anderson R. E. Tatham R. L. (2010). Multivariate Data Analysis, 7th ed. Upper Saddle River, HJ: Prentice Hall

[B37] Hayes J. R. (2012). Modeling and remodeling writing. Writ. Commun. 29, 369–388. 10.1177/0741088312451260

[B38] Hirose K. (2003). Comparing L1 and L2 organizational patterns in the argumentative writing of Japanese EFL students. J. Second Lang. Writ. 12, 181–209. 10.1016/S1060-3743(03)00015-8

[B39] Hooper D. Coughlan J. Mullen M. (2008). Evaluating model fit: a synthesis of the structural equation modelling literature. in Proceedings of the 7th European Conference on research methodology for business and management studies, ed. Brown A. (London, UK: Academic Publishing Limited), 195–200.

[B40] Hu L. Bentler P. M. (1999). Cutoff criteria for fit indexes in covariance structure analysis: conventional criteria versus new alternatives. Struct. Equ. Model. 6, 1–55. 10.1080/10705519909540118

[B41] Hutcheson G. D. Sofroniou N. (1999). The Multivariate Social Scientist: Introductory Statistics Using Generalized Linear Models. Thousand Oaks, CA: Sage.

[B42] Hyland K. Hyland F. (2019). Feedback in Second Language Writing: Contexts and Issues. Cambridge: Cambridge University Press.

[B43] Iwaniec J. (2020). Questionnaires: implications for effective implementation, in The Routledge Handbook of Research Methods in Applied Linguistics, eds McKinley J. Rose H. (New York, NY: Routledge) 327–335.

[B44] Jacobs H. L. Zinkgraf S. A. Wormuth D. R. Hartfiel V. F. Hughey J. B. (1981). Testing ESL Composition: A Practical Approach. Rowley, MA: Newbury House.

[B45] Jin Y. Zhang L. J. (2021). The dimensions of foreign language classroom enjoyment and their effect on foreign language achievement. Int. J. Biling. Educ. Biling. 24, 948–962. 10.1080/13670050.2018.1526253

[B46] Kaiser H. F. (1960). The application of electronic computers to factor analysis. Educ. Psychol. Meas. 20, 141–151. 10.1177/001316446002000116

[B47] Karlen Y. (2017). The development of a new instrument to assess metacognitive strategy knowledge about academic writing and its relation to self-regulated writing and writing performance. J. Writ. Res. 9, 61–86. 10.17239/jowr-2017.09.01.03

[B48] Kasper L. (1997). Assessing the metacognitive growth of ESL student writers. Tesl-Ej 3, 1–20.

[B49] Kline R. B. (2015). Principles and Practice of Structural Equation Modeling. 4th ed. New York, NY: Guilford.

[B50] Koriat A. (2000). The feeling of knowing: some metatheoretical implications for consciousness and control. Conscious. Cogn. 9, 149–171. 10.1006/ccog.2000.0433 10924234

[B51] Lee I. Mak P. (2018). Metacognition and metacognitive instruction in second language writing classrooms. TESOL Q. 52, 1085–1097. 10.1002/tesq.436

[B52] Marsh H. W. Balla J. R. McDonald R. P. (1988). Goodness-of-fit indexes in confirmatory factor analysis: the effect of sample size. Psychol. Bull. 103, 391–410. 10.1037/0033-2909.103.3.391

[B53] Negretti R. (2017). Calibrating genre: metacognitive judgments and rhetorical effectiveness in academic writing by L2 graduate students. Appl. Linguist. 38, 512–539.

[B54] Negretti R. McGrath L. (2018). Scaffolding genre knowledge and metacognition: insights from an L2 doctoral research writing course. J. Second Lang. Writ. 40, 12–31. 10.1016/j.jslw.2017.12.002

[B55] Nelson T. O. (1984). A comparison of current measures of the accuracy of feeling-of-knowing predictions. Psychol. Bull. 95, 109–133. 10.1037/0033-2909.95.1.109 6544431

[B56] Nelson T. O. (1996). Consciousness and metacognition. Am. Psychol. 51, 102–116. 10.1037/0003-066X.51.2.102

[B57] Norman E. Furnes B. (2016). The relationship between metacognitive experiences and learning: is there a difference between digital and non-digital study media? Comput. Human Behav. 54, 301–309. 10.1016/j.chb.2015.07.043

[B58] O'Malley J. M Chamot A. U. (1990). Learning Strategies in Second Language Acquisition. Cambridge: Cambridge University Press.

[B59] Pallant J. (2016). SPSS Survival Manual: A step by Step Guide to Data Analysis Using IBM SPSS. 6th ed. Crows Nest, NSW: Allen and Unwin

[B60] Papaleontious-Louca E. (2008). Metacognition and Theory of Mind. Newcastle: Cambridge Scholars Publishing.

[B61] Petrić B. Czárl B. (2003). Validating a writing strategy questionnaire. System 31, 187–215. 10.1016/S0346-251X(03)00020-4

[B62] Prior M. T. (2019). Elephants in the room: an “affective turn,” or just feeling our way? Mod. Lang. J. 103, 516–527. 10.1111/modl.12573

[B63] Raykov T. Marcoulides G. A. (2008). An introduction to applied multivariate analysis. New York, NY: Routledge.

[B64] Ruan Z. L. (2014). Metacognitive awareness of efl student writers in a Chinese ELT Context. Lang. Aware. 23, 76–91. 10.1080/09658416.2013.863901

[B65] Steiger J. H. (1990). Structural model evaluation and modification: an interval estimation approach. Multivariate Behav. Res. 25, 173–180. 10.1207/s15327906mbr2502_4 26794479

[B66] Tabachnick B. G. Fidell L. S. (2007). Using Multivariate Statistics. 5th ed. Hillsdale, NJ: Earlbaum.

[B67] Tarricone P. (2011). The Taxonomy of Metacognition. New York, NY: Psychology Press.

[B68] Teng F. (2020). The role of metacognitive knowledge and regulation in mediating university EFL learners' writing performance. Innov. Lang. Learn. Teach. 14, 436–450. 10.1080/17501229.2019.1615493

[B69] Teng L. S. Zhang L. J. (2016). A questionnaire-based validation of multidimensional models of self-regulated learning strategies. Modern Lang. J. 100, 674–701. 10.1111/modl.12339

[B70] Teng L. S. Zhang L. J. (2020). Empowering learners in the second/foreign language classroom: can self-regulated learning strategies-based writing instruction make a difference? J. Second Lang. Writ. 48, 1–14. 10.1016/j.jslw.2019.100701

[B71] Teng M. F. Zhang L. J. (2021). Development of children's metacognitive knowledge, reading, and writing in English as a foreign language: evidence from longitudinal data using multilevel models. Br. J. Educ. Psychol., e12413. 10.1111/bjep.12413 33694166

[B72] Tucker L. R. Lewis C. (1973). A reliability coefficient for maximum likelihood factor analysis. Psychometrika 38, 1–10. 10.1007/BF02291170

[B73] Victori M. (1999). An analysis of writing knowledge in EFL composing: a case study of two effective and two less effective writers. System 27, 537–555. 10.1016/S0346-251X(99)00049-4

[B74] Wei X. Zhang L. J. Zhang W. (2020). Associations of L1-to-L2 rhetorical transfer with L2 writers? perception of L2 writing difficulty and L2 writing proficiency. J. English Acad. Purp. 47, 100907, 1–14. 10.1016/j.jeap.2020.100907

[B75] Wenden A. L. (1998). Metacognitive knowledge and language learning. Appl. Linguist. 19, 515–537. 10.1093/applin/19.4.515

[B76] Winne P. H. Hadwin A. F. (1998). Studying as self-regulated learning, in Metacognition in Educational Theory and Practice, eds. Hacker D. J. Dunlosky J. Graesser A. C. (New York, NY: Routledge), 277–304.

[B77] Wirth J. Leutner D. (2008). Self-regulated learning as a competence: implications of theoretical models for assessment methods. Zeitschrift für Psychol. Psychol. 216, 102–110. 10.1027/0044-3409.216.2.102

[B78] Wu M. M. (2021). The social nature of second language metacognition. Asia-Pacific Educ. Res. 596:4. 10.1007/s40299-021-00596-4

[B79] Wu H. Y. (2006). Metacognitive experiences in college students' EFL writing. Foreign Lang. Their Teach., 28–30.

[B80] Yang L. Gao S. (2013). Beliefs and practices of Chinese university teachers in EFL writing instruction. Lang. Cult. Curric. 26, 128–145. 10.1080/07908318.2013.794817

[B81] Yeh H.-C. (2015). Facilitating metacognitive processes of academic genre-based writing using an online writing system. Comput. Assist. Lang. Learn. 28, 479–498. 10.1080/09588221.2014.881384

[B82] Yu X. (2020). Lexical features in argumentative writing across English writers from different language backgrounds. J. Second Lang. Stud. 3, 82–110. 10.1075/jsls.19024.yu

[B83] Zhang D. Zhang L. J. (2019). Metacognition and self-regulated learning (SRL) in second/foreign language teaching, in Second Handbook of English Language Teaching, ed. Gao X. (New York, NY: Springer International Publishing) 883–898.

[B84] Zhang L. Zhang L. J. (2013). Relationships between Chinese college test takers' strategy use and EFL reading test performance: a structural equation modeling approach. RELC J. 44, 35–57. 10.1177/0033688212463272

[B85] Zhang L. J. (2001). Awareness in reading: EFL students' metacognitive knowledge of reading strategies in an acquisition-poor environment. Lang. Aware. 10, 268–288. 10.1080/09658410108667039

[B86] Zhang L. J. (2008). Constructivist pedagogy in strategic reading instruction: Exploring pathways to learner development in the English as a second language (ESL) classroom. Instr. Sci. 36, 89–116. 10.1007/s11251-007-9025-6

[B87] Zhang L. J. (2010). A dynamic metacognitive systems account of Chinese university students' knowledge about EFL reading. TESOL Q. 44, 320–353. 10.5054/tq.2010.223352

[B88] Zhang L. J. Aryadoust V. Zhang D. (2016). Taking stock of the effects of strategies-based instruction on writing in Chinese and English in Singapore primary schools, in Quadrilingual Education in Singapore: Pedagogical Innovation in Language Education, eds. Silver R. E. Bokhorst-Heng W. (New York, NY: Springer), 103–126.

[B89] Zhang L. J. Qin T. L. (2018). Validating a Questionnaire on EFL Writers' Metacognitive Awareness of Writing Strategies in Multimedia Environments, in Metacognition in Language Learning and Teaching, eds Haukås Å. Bjørke C. Dypedahl M. (New York, NY: Routledge), 157–178. 10.4324/9781351049146-9

[B90] Zhang L. J. Thomas N. Qin T. L. (2019). Language learning strategy research in System: Looking back and looking forward. System 84, 87–92. 10.1016/j.system.2019.06.002

[B91] Zhang W. Zhang D. Zhang L. J. (2021b). Metacognitive instruction for sustainable learning: Learners' perceptions of task difficulty and use of metacognitive strategies in completing integrated speaking tasks. Sustainability 13, 6275, 1–21. 10.3390/su13116275

[B92] Zhang W. Zhang L. J. Wilson A. J. (2021a). Supporting learner success: revisiting strategic competence through developing an inventory for computer-assisted speaking assessment. Front. Psychol. 12, 1–14. 10.3389/fpsyg.2021.689581 34163415 PMC8215541

[B93] Zhang L. J. (2002). Exploring EFL reading as a metacognitive experience: reader awareness and reading performance. Asian J. Eng. Lang. Teach. 12, 69–94.

