# Supplementary material for: Investigating Students' Metacognitive Experiences: Insights From the English as a Foreign Language Learners' Writing Metacognitive Experiences Questionnaire (EFLLWMEQ)
Source: Front Psychol. 2021 Aug 30;12:744842. doi: 10.3389/fpsyg.2021.744842 (PMC8437130; doi:10.3389/fpsyg.2021.744842)
Supplement: Supplementary file 1 [file Data_Sheet_1.docx]

# APPENDIX A

**EFL Learners’ Writing Metacognitive Experiences Questionnaire (EFLLWMEQ)**

**I. Participants Demographic Information**

*In this part, please provide the following information by ticking (√) in the box or writing down your responses in the space. Please try not to leave out any of the items.*

1 Student Code: Gender: Male □ Female □ Age: Major: ____

2 Years of Learning English:

3 English proficiency reflected by test: (*You can choose more than one item*)

CET4 □ CET6 □ TEM4 □ TEM8 □ IELTS□ (Overall Score ) TOFEL□ (Overall Score ) Others: (Overall Score )

4 Overseas experience: Have you ever been to an English-speaking country for studying, travelling or other purposes?

Yes □ No □

If yes, please state how long: and for what purpose:

**II*.* EFL Learners’ Writing Metacognitive Experiences**

The following statements are about what you may or may not have experienced in learning to write in English. Please give your answers honestly to guarantee the success of the survey.

*In the following section, we would like you to tell us how much you agree or disagree with the following statements by ticking (√) a number from 1 to 6. Please do not leave out any of the items.*

| Strongly **disagree** | Disagree | Slightly disagree | Slightly agree | Agree | Strongly **agree** |
| --- | --- | --- | --- | --- | --- |
| 1 | 2 | 3 | 4 | 5 | 6 |

| 1. I am interested in writing. | 1 2 3 4 5 6 |
| --- | --- |
| 5. I finish the writing task as expected. | 1 2 3 4 5 6 |
| 7. I feel confident about myself as a writer. | 1 2 3 4 5 6 |
| 8. I check my grammatical errors. | 1 2 3 4 5 6 |
| 1. I re-organise my ideas. | 1 2 3 4 5 6 |
| 10. I check my spelling. | 1 2 3 4 5 6 |
| 11. I check my sentence structures. | 1 2 3 4 5 6 |
| 1. I ensure the first and last sentences are strong enough to explain my meaning. | 1 2 3 4 5 6 |
| 1. I learn/write more if I am interested in this writing topic. | 1 2 3 4 5 6 |
| 1. I use what I have learned from my English courses. | 1 2 3 4 5 6 |
| 16. I am satisfied with my writing. 1 2 3 4 5 6 | |
| 1. I check if I finish these writing tasks on time. | 1 2 3 4 5 6 |
| 1. I pay attention to grammar use in my writing. | 1 2 3 4 5 6 |
| 22. I pay attention to vocabulary use in my writing. | 1 2 3 4 5 6 |
| 23. I pay attention to sentence structures in my writing. | 1 2 3 4 5 6 |
| 24. I pay attention to logical reasoning in my writing. | 1 2 3 4 5 6 |

**APPENDIX B**

**Writing Prompt**

Our writing ability is very important to all of us, but different people have different views on its importance. Do you think writing ability is important? Give your reasons to support your argument.
